# Association between hyperCKemia and axonal degeneration in Guillain–Barré syndrome

**DOI:** 10.1186/s12883-023-03104-x

**Published:** 2023-03-02

**Authors:** Eun Kyoung Lee, Sooyoung Kim, Nathan Jo, Eunhee Sohn

**Affiliations:** 1 Department of Neurology, Chungnam National University Sejong Hospital, Sejong, Republic of Korea; 2 Department of Neurology, Chungnam National University Hospital, 282 Moonhwa-Ro, Jung-Gu, Daejeon, 35015 Republic of Korea

**Keywords:** Guillain–Barré syndrome, Creatine kinase, Axonal degeneration, Reversible conduction failure

## Abstract

**Background:**

Elevated serum creatine kinase (CK) levels have been reported in patients with Guillain–Barré syndrome (GBS), more frequently in patients with acute motor axonal neuropathy (AMAN) than in those with acute inflammatory demyelinating polyneuropathy (AIDP). However, some patients with AMAN show reversible conduction failure (RCF), characterized by rapid recovery without axonal degeneration. The present study tested the hypothesis that hyperCKemia is associated with axonal degeneration in GBS, regardless of the subtype.

**Methods:**

We retrospectively enrolled 54 patients with AIDP or AMAN whose serum CK levels were measured within 4 weeks from symptom onset between January 2011 and January 2021. We divided them into hyperCKemia (serum CK ≥ 200 IU/L) and normal CK (serum CK < 200 IU/L) groups. Patients were further classified into axonal degeneration and RCF groups based on more than two nerve conduction studies. The clinical features and frequency of axonal degeneration and RCF were compared between groups.

**Results:**

Clinical characteristics were similar in the hyperCKemia and normal CK groups. Compared with that in the RCF subgroup, the frequency of hyperCKemia was significantly higher in the axonal degeneration group (*p* = 0.007). Patients with normal serum CK levels showed better clinical prognosis, evaluated by the Hughes score at 6 months from admission (*p* = 0.037).

**Conclusion:**

HyperCKemia is associated with axonal degeneration in GBS, regardless of the electrophysiological subtype. HyperCKemia within 4 weeks from symptom onset might be a marker of axonal degeneration and poor prognosis in GBS. Serial nerve conduction studies and serum CK measurements will help clinicians understand the pathophysiology of GBS.

## Background

Guillain–Barré syndrome (GBS) is an immune-mediated inflammatory polyradiculoneuropathy that can be divided into acute inflammatory demyelinating polyneuropathy (AIDP) and acute motor axonal neuropathy (AMAN) on the basis of electrodiagnostic tests [1–3]. AMAN has clinical features similar to those of AIDP, and it is mainly characterized by decreased or lost amplitude of compound muscle action potentials (CMAPs; < 80% of the lower limit of normal) in electrodiagnostic tests. Some patients with AMAN show conduction block without temporal dispersion and recover within a short period of time; this is called reversible conduction failure (RCF) [4–6]. The main pathophysiology of AMAN is the immune response at the node of Ranvier, which is induced by antiganglioside antibodies. It has been postulated that an aborted immune attack before progression to axonal degeneration results in transient conduction failure with rapid recovery [7–10].

Creatine kinase (CK) plays an important role in the catalysis of creatine and adenosine triphosphate (ATP) to adenosine diphosphate (ADP) in muscle, and it leaks from myocytes into the blood even under normal conditions [11, 12]. CK elevation demonstrates active degeneration of myocytes and is commonly seen in myopathies such as rhabdomyolysis and inflammatory myositis. Some neurogenic diseases such as motor neuron disease and GBS also cause elevated serum CK levels. The underlying mechanism of hyperCKemia in patients with GBS and the clinical characteristics of GBS patients with elevated serum CK levels remain unclear [13].

A recent study showed that transient hyperCKemia was associated with the non-demyelinating subtype of GBS [14]. The authors categorized the patients into axonal, demyelinating, equivocal, and normal subgroups according to electrodiagnostic tests based on Rajabally’s modified criteria [14]. However, they did not consider RCF in axonal GBS, which is quite different from AMAN with axonal degeneration in terms of pathophysiology and prognosis [14].

The aim of the present study was to determine the clinical characteristics of GBS patients with hyperCKemia and compare them with those of GBS patients with normal serum CK levels. We postulated that axonal degeneration is associated with hyperCKemia in GBS, regardless of the electrophysiological subtype. We classified the patients into axonal degeneration and RCF groups to identify the relationship between axonal damage and serum CK levels in GBS patients.

## Methods

### Patients and clinical data

We retrospectively evaluated 97 patients with GBS who were admitted to Chungnam National University Hospital from January 2011 to January 2021. From these patients, we enrolled those with generalized weakness who could be categorized under AIDP or AMAN on the basis of more than two serial electrodiagnostic tests performed within 1 month from symptom onset. Twenty-seven patients were excluded because of focal weakness; these included 19 patients with Miller Fisher syndrome and eight with the pharyngeal–cervical–brachial variant. Six patients were excluded because their electrodiagnostic tests were incompatible with AIDP or AMAN. Another patient was lost to follow-up. Among the remaining 63 patients, 54 whose serum CK levels were measured within 4 weeks from symptom onset were eventually enrolled.

The patients were divided into two groups. Those with a serum CK level of ≥ 200 IU/L were classified in the hyperCKemia group, whereas those with a serum CK level of < 200 IU/L were classified in the normal CK group, based on a previous study [13].

Clinical variables such as age, sex, preceding infection of the respiratory or gastrointestinal tract, bulbar symptoms, facial palsy, and sensory symptoms were reviewed. Periods from symptom onset to the nadir point, symptom onset to treatment initiation, and symptom onset to serum CK level measurement were recorded. The total protein level in the cerebrospinal fluid (CSF) and the status of antiganglioside antibodies (IgG & IgM antibodies against GM1, GD1b, and GQ1b) were also assessed.

Current statin use, history of trauma or surgery, excessive exercise, and renal failure were investigated as factors that could affect serum CK elevation. All 54 patients were treated with intravenous immunoglobulin therapy (0.4 g/kg/day) for 5 days, and none of them underwent plasmapheresis.

Disabilities were evaluated using the Hughes score: grade 0, healthy; grade 1, minor signs and symptoms, able to run; grade 2, able to walk independently; grade 3, able to walk with a walker or support; grade 4, bed- or chair-bound; grade 5, assisted respiration required for at least part of the day; and grade 6, dead. The Hughes score was evaluated at the nadir point and 1 and 6 months after admission. A Hughes score of < 3 at 6 months was considered to represent a good prognosis, and we compared the frequency of patients with a good prognosis between the hyperCKemia and normal CK groups.

This study was approved by the Institutional Review Board of Chungnam National University Hospital (approval number: 2019–09-055–002). All procedures were performed in accordance with the principles of the Declaration of Helsinki. The informed consent was waived because of the retrospective nature of the study.

### Electrophysiological examinations

Motor and sensory nerve conduction studies were performed using standard techniques (Viking Select ™, Madison, WI, USA). Motor nerve conduction examinations were performed for the median, ulnar, peroneal, and tibial nerves. Measured parameters included the CMAP amplitude, distal motor latency, motor nerve conduction velocity, and minimal F-wave latency. Orthodromic sensory nerve conduction examinations were performed for the median, ulnar, superficial peroneal, and sural nerves. Examinations were performed immediately, 1 week, and 1 month after admission.

### Classification of GBS subgroups

We analyzed more than two serial nerve conduction studies within a month from symptom onset. Patients were classified into the ADIP or AMAN group according to the electrodiagnostic criteria described by Uncini et al. [15, 16]; they were subsequently reevaluated if they showed RCF or axonal degeneration in electrodiagnostic tests.

AIDP was diagnosed in patients with electrodiagnostic features of demyelination in at least two nerves, such as prolonged distal latency that was > 130% of the upper limit of normal (ULN), motor velocity slowing that was < 70% of the lower limit of normal (LLN), prolonged F-wave latency that was > 120% of ULN, a distal CMAP duration that was > 120% of ULN, or a proximal CMAP to distal CMAP duration ratio of > 130%. AMAN was diagnosed in patients who showed no evidence of demyelination in all serial nerve conduction studies, with decreased distal CMAP amplitudes below 80% of LLN or a proximal CMAP to distal CMAP amplitude ratio of < 0.7 in at least two nerves in the first nerve conduction study and evidence of axonal degeneration or RCF in the two nerves in the second nerve conduction study [16].

RCF was defined by the following: a distal CMAP amplitude that was > 150% of that in the first nerve conduction study, without an increased distal CMAP duration (≤ 120%) in the second study; or an increase of 0.2 in the proximal CMAP to distal CMAP amplitude ratio, without abnormal temporal dispersion (proximal CMAP/distal CMAP duration ratio: ≤ 130%) [16]. Axonal degeneration was defined when proximal and distal CMAP amplitudes in serial tests showed a continuous decrease without temporal dispersion, with no recovery at 1 month relative to the values in previous electrodiagnostic tests. The criteria for RCF and axonal degeneration were applied to all enrolled patients, regardless of AIDP or AMAN [16].

Based on this classification, we divided the patents into four subgroups: AIDP with RCF, AIDP without RCF, AMAN with RCF, and AMAN without RCF. Then, we divided the patients into three groups according to serial changes in CMAP: RCF, axonal degeneration, and undetermined [16, 17]. The RCF and axonal degeneration groups were compared to determine the relationship between serum CK levels and axonal degeneration.

### Statistical analysis

All statistical analyses were performed using SPSS version 20.0 (SPSS Inc., Chicago, IL, USA). Summary statistics of demographic and clinical characteristics and other collected variables for the hyperCKemia and normal CK groups were presented as proportions, means, standard deviations, and ranges. The independent t-test, Pearson’s chi-square test, Fisher’s exact test, and Mann–Whitney U test to were performed to compare the statistical significance of the hyperCKemia and normal CK groups. All statistical analyzes were performed at a significance level *p*-value of 0.05.

## Results

The mean age of the 54 patients was 58.3 ± 13.67 (ranged, 26–86) years, and there were 34 men and 20 women. There were 18 patients (33.3%) in the hyperCKemia group, and the mean CK level was 385.9 ± 185.0 (range, 200–802 IU/L). There were no significant differences between the hyperCKemia and normal CK groups in terms of age, sex, preceding infection, symptoms of GBS, total protein level in CSF, periods from symptom onset to the nadir point and from symptom onset to treatment initiation, and the status of antiganglioside antibodies. The mean Hughes scores at the nadir point and 1 and 6 months after admission were 3.9 ± 1.0, 2.8 ± 1.4, and 1.6 ± 1.6, respectively, with no significant differences (Table 1).

**Table 1:** Clinical characteristics of patients with Guillain-Barré syndrome showing hyperCKemia or normal CK levels.

	HyperCKemia (*n* = 18)	Normal CK levels (*n* = 36)	*p*-value
Male/female	12/6	22/14	0.69
Age (years)	56.1 ± 12.1	59.4 ± 14.4	0.38
Statin use (%)	4 (22.2)	10 (27.7)	0.14
Preceding infection (%)			0.32
URI	5 (27.8)	11 (30.6)	
Diarrhea	8 (44.4)	17 (47.2)	
URI & diarrhea	2 (11.1)	0	
Operation	0	1 (2.8)	
None	3 (16.7)	8 (22.2)	
Absence of sensory symptom (%)	6 (33.3)	8 (22.2)	0.28
Presence of bulbar symptoms (%)	4 (22.2)	11 (30.5)	0.53
Presence of facial palsy (%)	2 (11.1)	6 (16.7)	0.60
CSF total protein level (mg/dl) (range)	50.3 ± 23.5 (18–140)	62.2 ± 36.2 (30–94)	0.52
Periods from onset to nadir (days)	5.8 ± 2.7	7.3 ± 3.0	0.08
Periods from onset to IVIG treatment (days)	3.6 ± 2.6	6.9 ± 10.2	0.08
Serum CK (IU/L) (range)	385.9 ± 185.0 (200–802)	110.1 ± 48.6 (31–183)	0.00*
Periods from onset to CK measurement (days) (range)	3.5 ± 2.7 (1–10)	5.4 ± 5.2 (0–20)	0.34
Number of antiganglioside antibodies (%)	3/14 (21.4)	2/27 (7.4)	0.20
Hughes score at nadir	3.9 ± 1.0 (1–5)	3.5 ± 1.1 (1–5)	0.29
Hughes score at 1 month	2.8 ± 1.4 (1–5)	2.6 ± 1.2 (1–5)	0.60
Hughes score at 6 months	1.6 ± 1.6 (0–4)	1.1 ± 1.3 (0–4)	0.26

Values are presented as mean ± standard deviation (minimum–maximum) or number (%)

*Abbreviations*: *CK* Creatine kinase, *Uri* Upper respiratory infection, *CSF* Cerebrospinal fluid, *NCS* Nerve conduction study, *IVIG* Intravenous immunoglobulin

^*^Statically significant (*p* < 0.05)

None of the patients had a history of trauma, excessive exercise, or renal failure, and there were no differences in the proportion of statin users (22.2% in the hyperCKemia group; 27.7% in the normal CK group). The period from symptom onset to CK level measurement was not significantly different between the hyperCKemia and normal CK groups (Table 1).

In fifty-four patients, ten patients were classified as AMAN with RCF, twenty-two patients as AMAN without RCF, two patients as AIDP with RCF, and twenty patients as AIDP without RCF group (Table 2). Fifteen patients in the axonal degeneration group who were showed a continuous decrease of CMAP amplitudes without temporal dispersion included eleven patients in AMAN without RCF group and four patients in AIDP without RCF group (Table 2).

**Table 2:** Frequencies of hyperCKemia and normal CK levels according to the electrophysiological subtype of Guillain-Barré syndrome

Electrophysiological subtypes	HyperCKemia (%)	Normal CK levels (%)	*p*-value
AMAN and AIDP (*n* = 54)			0.098
AMAN with RCF	1 (5.6)	9 (25.0)	
AMAN without RCF	11 (61.1)	11 (30.6)	
AIDP with RCF	0 (0.0)	2 (5.6)	
AIDP without RCF	6 (33.3)	14 (38.9)	
RCF and axonal degeneration (*n* = 27)			0.007*
RCF	1 (10.0)	11 (64.7)	
Axonal degeneration	9 (90.0)	6 (35.3)	

Values are presented as number (%)

*Abbreviations*: *CK* Creatine kinase, *AMAN* Acute motor axonal neuropathy, *RCF* Reversible conduction failure, *AIDP* Acute inflammatory demyelinating polyneuropathy

^*^Statically significant (p < 0.05)

The hyperCKemia group included more patients with AMAN/AIDP without RCF, and one of 12 patients with AMAN/AIDP with RCF belonged to the hyperCKemia group. There were no significant differences between groups according to the RCF status (Table 2). HyperCKemia was more frequent in the axonal degeneration subgroup, whereas normal CK levels were more frequent in the RCF group, with a significant difference between groups (*p* = 0.007; Table 2).

**Fig. 1:**
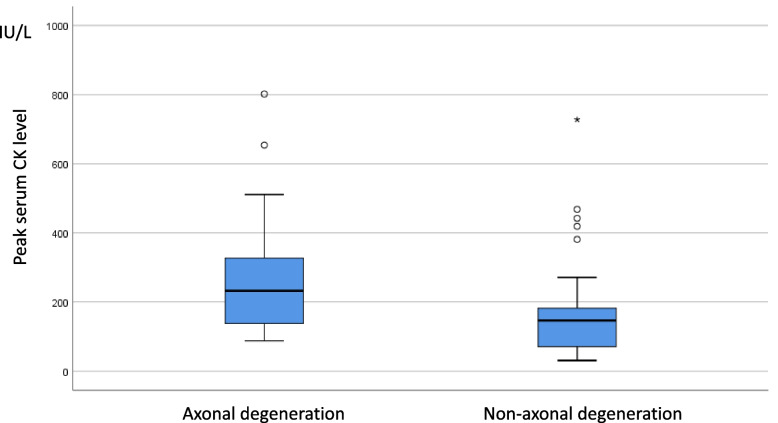
Serum CK levels between the axonal degeneration group and the non-axonal degeneration group. Abbreviation: CK, creatine kinase.

In the comparison of serum CK levels between the axonal degeneration group and non-axonal degeneration group, the mean serum CK level of axonal degeneration group was 286.60 ± 212.81 IU/L, which was significantly higher than of the non-axonal degeneration which was 169.54 ± 144.57 IU/L (*p* = 0.024; Fig. 1).

According to the Hughes score at 6 months, the normal CK group showed a significantly better prognosis than did the hyperCKemia group (86.1% in the normal CK group, 61.1% in the hyperCKemia group; *p* = 0.037; Table 3).

**Table 3:** Comparison of prognosis between patients with hyperCKemia and those with normal CK levels in a cohort of patients with Guillain-Barré syndrome

	HyperCKemia (*n* = 18), (%)	Normal CK levels (*n* = 36), (%)	*p*-value
Hughes score at 6 months: < 3	11 (61.1)	31 (86.1)	0.037*
Hughes score at 6 months: ≥ 3	7 (38.9)	5 (13.9)	

Values are presented as number (%)

*Abbreviations*: *CK* Creatine kinase

^*^Statically significant (*p* < 0.05)

## Discussion/Conclusions

In the present study of GBS patients (AIDP/AMAN), elevated serum CK levels within 4 weeks from symptom onset were found in 18 of 54 patients (33.3%) with generalized weakness. Elevated serum CK levels were significantly more frequent in patients with axonal degeneration, whereas normal serum CK levels were more frequent in patients with RCF. Patients with normal serum CK levels within 4 weeks from symptom onset showed a better clinical prognosis according to the Hughes scores at 6 months from admission.

Elevated serum CK levels were not uncommon in the present study. Limited studies have evaluated elevated serum CK levels in GBS; these included case series and a few retrospective studies [13, 14]. Studies have reported transient elevations in serum CK levels of 16.7% [14] and 27% [13] in GBS patients. Elevated serum CK levels would be more frequent in patients with GBS, especially those with generalized weakness, and further systematic prospective studies are needed.

We found that elevated serum CK levels were associated with axonal degeneration and poor prognosis regardless of the electrophysiological subtype of GBS. One recent study indicated that elevated serum CK levels represent AMAN with axonal degeneration or RCF, but not AIDP [13].

The authors categorized patients under “AIDP or AMAN” using the criteria of Ho et. al. and designated their condition as “unclassified” if the electrodiagnostic criteria did not fulfil the criteria for AIDP or AMAN. RCF was defined by electrodiagnosis of the “AIDP pattern” without sensory nerve conduction abnormalities, or those who were designated as “unclassified.” Thirteen “unclassified” patients were included among the 28 patients showing AMAN with RCF in that study. Therefore, the “AMAN with RCF” group included “unclassified” patients as well as patients showing AMAN with RCF, which may not represent the proper form of RCF. In addition, follow-up electrodiagnostic tests were conducted for 34 of 51 patients (66.7%), which made precise definition of RCF difficult. The classical electrodiagnostic criteria proposed by Ho et al. or Rajabally et al. are based on a single electrodiagnostic study. Several studies have reported that the classical electrodiagnostic criteria occasionally misdiagnose GBS subgroups, and that the classification changes after serial electrodiagnostic tests [15]. Uncini et al. [15, 16] defined RCF through two or more follow-up electrodiagnostic studies and emphasized the importance of serial electrodiagnostic tests for precise subgrouping of GBS. We classified our patients according to the criteria of Uncini et al. [15, 16] and found patients with RCF in both the AMAN (31.3%) and AIDP (9.1%) groups. Uncini et al. [15, 16] reported that RCF was present in 46.6% patients with axonal GBS and 15% patients with AIDP, similar to the results in the present study [15]. Another recent study also reported that RCF in AIDP can be observed and is comparable with AMAN with RCF in terms of clinical features [17].

The mechanism of CK elevation in GBS patients is not fully understood. It has been suggested that rapid and extensive denervation of motor nerve terminals might cause hyperexcitability of muscles and subsequent intramuscular CK release; this has been described in previous studies as an axonal pathology [11, 14]. It is widely accepted that in AMAN there may be Wallerian-like degeneration leading to muscle denervation, and which can be the basis for serum CK elevation and paralysis sequelae [18]. Axonal degeneration may be necessary pathophysiological process for serum CK elevation given that not just AMAN patients but also AIDP patients [4]. A recent study pointed to inflammatory edema of nerve trunks causing ischemic conduction failure, which in the ensuing days can lead to Wallerian-like degeneration [19, 20].

The present study showed a relationship between hyperCKemia and axonal degeneration, thus supporting this postulated hypothesis. A relationship with rhabdomyolysis caused by antecedent infection has been suggested as another possible mechanism. However, this mechanism is less likely because the serum CK level was not as high as that observed in rhabdomyolysis in previous studies and this study [21].

To the best of our knowledge, no previous report has focused on the association between hyperCKemia and RCF in GBS. In this study, we defined RCF using the criteria of Uncini et al. [15, 16]. In addition, a good correlation between elevated serum CK levels and axonal degeneration was shown by using serial electrodiagnostic analysis. According to the present results, the classification of AMAN or AIDP using current electrodiagnostic criteria is considered to have little correlation with the elevation of serum CK levels. This is because AIDP may be accompanied by axonal degeneration as well as demyelination; therefore, it would be difficult to suggest that the demyelinating pattern in electrodiagnostic tests indicates a normal CK level. In addition, if the serum CK level measured within 4 weeks from symptom onset is elevated, regardless of AIDP or AMAN, it could be an indicator of axonal degeneration. Serial nerve conduction studies and the measurement of serum CK levels will help clinicians understand the pathophysiology of GBS.

This study had several limitations. First, because of the retrospective nature of the study in a tertiary center, the sample size was small, and clinical features, including sensory symptoms and predisposing factors, could be insufficient. Because of the retrospective design, we could not describe sensory symptoms in detail and could not exclude all factors that would affect serum CK levels. Second, the serum CK levels were measured only once. Planned serial serum CK measurements would help exclude other causes of hyperCKemia. Third, the prevalence of AMAN in Eastern countries such as Korea and Japan is higher than that in Western countries. Further studies involving various populations are required. Finally, the sensitivity of antiganglioside antibody testing was low, and the antiganglioside antibody test was not performed for all patients. Antiganglioside antibodies represent one of the major pathogenic mechanisms of AMAN, and they would be associated with hyperCKemia in GBS.

In conclusion, the findings of this study suggest that hyperCKemia is associated with axonal degeneration in GBS, regardless of the electrophysiological subtype. HyperCKemia within 4 weeks from symptom onset might be a marker of axonal degeneration and poor prognosis in GBS. Serial nerve conduction studies and serum CK measurements will help clinicians understand the pathophysiology of GBS.

## Data Availability

Data will be made available upon reasonable request to the corresponding author.

## References

[1] Yuki NHH (2012). Guillain-Barré syndrome. N Engl J Med.

[2] Lewis RA (2018). Electrophysiologic lessons from the European multicenter study of Guillain-Barré syndrome subtype diagnosis. Muscle Nerve.

[3] Kaida K (2019). Guillain-Barré Syndrome. Adv Exp Med Biol.

[4] Uncini A, Kuwabara S (2012). Electrodiagnostic criteria for Guillain-Barrè syndrome: a critical revision and the need for an update. Clin Neurophysiol.

[5] Van den Bergh PYK, Pieret F, Woodard JL, Attarian S, Grapperon AM, Nicolas G, et al. Guillain–Barrè syndrome subtype diagnosis: A prospective multicentric European study. Muscle Nerve. 2018. DOI: 10.1002/mus.26056. Online ahead of print. 10.1002/mus.26056 29315669

[6] Kuwabara S, Asahina M, Koga M, Mori M, Yuki N, Hattori T (1998). Two patterns of clinical recovery in Guillain-Barré syndrome with IgG anti-IgM antibody. Neurology.

[7] Wanleenuwat P, Iwanowski P, Kozubski W (2020). Antiganglioside antibodies in neurological diseases. J Neurol Sci.

[8] Uncini A, Vallat JM (2018). Autoimmune nodo-paranodopathies of peripheral nerve: the concept is gaining ground. J Neurol Neurosurg Psychiatry.

[9] Uncini A, Mathis S, Vallat JM (2022). New classification of autoimmune neuropathies based on target antigens and involved domains of myelinated fibres. J Neurol Neurosurg Psychiatry.

[10] Vallat JM, Magy L, Corcia P, Boulesteix JM, Uncini A, Mathis S (2020). Ultrastructural Lesions of Nodo-Paranodopathies in Peripheral Neuropathies. J Neuropathol Exp Neurol.

[11] Satoh JOK, Kishi T, Nagayama S, Kuroda Y (2000). Cramping pain and prolonged elevation of serum creatine kinase levels in a patient with Guillain-Barré syndrome following campylobacter jejuni enteritis. Eur J Neurol.

[12] Silvestri NJ, Wolfe GI (2013). Asymptomatic/pauci-symptomatic creatine kinase elevations (hyperckemia). Muscle Nerve.

[13] Hosokawa T, Nakajima H, Sawai T, Nakamura Y, Sano E, Tsukahara A (2020). Clinical features of Guillain-Barré syndrome patients with elevated serum creatine kinase levels. BMC Neurol.

[14] Choi SJ, Hong YH, Kim JS, Shin JY, Sung JJ (2020). HyperCKemia in Guillain-Barré Syndrome. Eur Neurol.

[15] Uncini A, Kuwabara S (2018). The electrodiagnosis of Guillain-Barré syndrome subtypes: Where do we stand?. Clin Neurophysiol.

[16] Uncini A, Ippoliti L, Shahrizaila N, Sekiguchi Y, Kuwabara S (2017). Optimizing the electrodiagnostic accuracy in Guillain-Barré syndrome subtypes: Criteria sets and sparse linear discriminant analysis. Clin Neurophysiol.

[17] Kim S, Lee EK, Sohn E. Reversible Conduction Failure in Acute Inflammatory Demyelinating Polyneuropathy. Sci Rep. 2022;12(1):18562. 10.1038/s41598-022-19547-0 PMC9633831 36329046

[18] Tamura N, Kuwabara S, Misawa S, Kanai K, Nakata M, Sawai S (2007). Time course of axonal regeneration in acute motor axonal neuropathy. Muscle Nerve.

[19] Nedkova V, Gutiérrez-Gutiérrez G, Navacerrada-Barrero FJ, Berciano J, Casasnovas C (2021). Re-evaluating the accuracy of optimized electrodiagnostic criteria in very early Guillain-Barré syndrome: a sequential study. Acta Neurol Belg.

[20] Berciano J (2020). Inflammatory oedema of nerve trunks may be pathogenic in very early Guillain-Barré syndrome. Acta Neurol Belg.

[21] Scott A, Duncan R, Henderson L, Jamal G, Kennedy P (1991). Acute rhabdomyolysis associated with atypical Guillain-Barré syndrome. Postgrad Medical J.

